# Hellinger Information Matrix and Hellinger Priors

**DOI:** 10.3390/e25020344

**Published:** 2023-02-13

**Authors:** Arkady Shemyakin

**Affiliations:** Mathematics Department, University of St. Thomas, 2115 Summit Ave, St. Paul, MN 55105, USA; a9shemyakin@stthomas.edu

**Keywords:** Hellinger information, Hellinger priors, Bayes risk

## Abstract

Hellinger information as a local characteristic of parametric distribution families was first introduced in 2011. It is related to the much older concept of the Hellinger distance between two points in a parametric set. Under certain regularity conditions, the local behavior of the Hellinger distance is closely connected to Fisher information and the geometry of Riemann manifolds. Nonregular distributions (non-differentiable distribution densities, undefined Fisher information or denisities with support depending on the parameter), including uniform, require using analogues or extensions of Fisher information. Hellinger information may serve to construct information inequalities of the Cramer–Rao type, extending the lower bounds of the Bayes risk to the nonregular case. A construction of non-informative priors based on Hellinger information was also suggested by the author in 2011. Hellinger priors extend the Jeffreys rule to nonregular cases. For many examples, they are identical or close to the reference priors or probability matching priors. Most of the paper was dedicated to the one-dimensional case, but the matrix definition of Hellinger information was also introduced for higher dimensions. Conditions of existence and the nonnegative definite property of Hellinger information matrix were not discussed. Hellinger information for the vector parameter was applied by Yin et al. to problems of optimal experimental design. A special class of parametric problems was considered, requiring the directional definition of Hellinger information, but not a full construction of Hellinger information matrix. In the present paper, a general definition, the existence and nonnegative definite property of Hellinger information matrix is considered for nonregular settings.

## 1. Introduction

Information geometry plays an important role in parametrical statistical analysis. Fisher information is the most common information measure that is instrumental in the construction of lower bounds for quadratic risk (information inequalities of Cramer–Rao type), optimal experimental designs (E-optimality) and noninformative priors in Bayesian analysis (Jeffreys prior). These applications of Fisher information require certain regularity conditions on the distributions of the parametric family, which include the existence and integrability of the partial derivatives of the distribution density function with respect to the components of vector parameter and independence of the density support on the parameter. If these regularity conditions are not satisfied, Cramer–Rao lower bounds might be violated, and Jeffreys prior might not be defined.

There exist a number of ways to define information quantities (for the scalar parameter case) and matrices (for the vector parameter case) in the nonregular cases when Fisher information might not exist. One of such suggestions is the Wasserstein information matrix [1], which has been recently applied to the construction of objective priors [2]. The Wasserstein matrix does not require the differentiablity of the distribution density function, but it cannot be extended to the case of discontinuous densities. This latter case requires a more general definition of information.

Our approach is based on analyzing the local behavior of parametric sets using finite differences of pdf values at two adjacent points instead of derivatives at a point, which allows us to include differentiable densities as a special case but also to treat non-differentiable densities including jumps and other types of nonregular behavior (for classification of nonregularities, see [3]). A logical approach is to use (in lieu of Fisher information) the Hellinger information closely related to the definition of Hellinger distance between adjacent points of the parametric set.

Hellinger information for the case of scalar parameter was first defined in [4] and suggested for the construction of noninformative Hellinger priors. Section 2 is dedicated to the revision of this definition and the relationship of Hellinger information to the information inequalities for the scalar parameter proven in [5,6]. It contains some examples of noninformative Hellinger priors comparing the priors obtained in [4] with more recent results.

Lin et al. [7] extended the definition of Hellinger information to a special multiparameter case, where all components of a parameter expose the same type of nonregularity. This is effective in the resolution of some optimization problems in experimental design. However, the most interesting patterns of local behavior of Hellinger distance bringing about differences in the behavior of matrix lower bounds of the quadratic risk and multidimensional noninformative priors are observed when the parametric distribution family has different orders of nonregularity [3] for different components of the vector parameter. Thus, the main challenge in the construction of a Hellinger information matrix in the general case consists in the necessity to consider different magnitudes of increments in different directions of the vector parametric space.

A general definition of the Hellinger information matrix was attempted in [6] as related to information inequalities and in [4] as related to noninformative priors. Important questions were left out, such as the conditions of Hellinger information matrix being positive definite and the existence of non-trivial matrix lower bounds for the quadratic risk in case of the vector parameter. These questions are addressed in Section 3 of the paper. The main results are formulated, and several new examples are considered. General conclusions and possible future directions of study are discussed in Section 4.

## 2. Hellinger Information for Scalar Parameter

In this section, we address the case of probability measures parametrized by a single parameter. We provide necessary definitions of information measures along with the discussion of their properties, including the new definition of Hellinger information. Then, we consider the applications of Hellinger information to the information inequalities of the Cramér–Frechet–Rao type, the construction of objective priors, and problems of optimal design.

Definition (2) is modified from [8]. Inequality (5) was obtained in [5]. Examples in Section 2.3 are modified from [4].

### 2.1. Definitions

A family of probability measures $\left\{P_{\theta },\theta \in \Theta \subset R\right\}$ is defined on a measurable space (X,B) so that all the measures from the family are absolutely continuous with respect to some σ-finite measure λ on B. The square of the Hellinger distance between any two parameter values can be defined in terms of densities $p\left(x;\theta \right)=\frac{dP_{\theta }}{d\lambda }$ as

(1) $$h^{2}\left(\theta_{1},\theta_{2}\right)=\int_{X}{\left(\sqrt{p(x;\theta_{1})}-\sqrt{p(x;\theta_{2})}\right)}^{2}\lambda \left(dx\right).$$

This definition of the Hellinger distance (also known as Hellinger–Bhattacharyya distance) in its modern form was given in [9]. We use this definition to construct a new information measure. If for almost all θ from Θ (with regard to measure λ) there exists an α∈(0,2] (**index of regularity**) such that

(2) $$\lim_{\epsilon \to 0}\frac{h^{2/\alpha }\left(\theta ,\theta +\epsilon \right)}{{\left|\epsilon \right|}^{2}}=J\left(\theta \right),$$

we define **Hellinger information** at a point θ as J(θ). The index of regularity is related to the local behavior of the density p(x;θ). Using the classification of [3], 1<α<2 corresponds to singularities of the first and the second type, α=1 to densities with jumps, and 0<α<1 to singularities of the third type.

Notice that in the regular situations classified in [3] (p(x;θ) is twice continuously differentiable with respect to θ for almost all x∈X with respect to λ, the density support {x:p(x;θ)>0} does not depend on parameter θ, Fisher information

(3) $$I\left(\theta \right)=E_{\theta }{\left(\frac{d}{d\theta }\log p\left(x;\theta \right)\right)}^{2}=\int_{X}{\left(\frac{d}{d\theta }p\left(x;\theta \right)\right)}^{2}/p\left(x;\theta \right)\lambda \left(dx\right)$$

is continuous, strictly positive and finite for almost all θ from Θ), it is true that $\alpha =2,J\left(\theta \right)=\frac{1}{4}I\left(\theta \right)$. Under the regularity conditions above, the score function $\frac{d}{d\theta }\log p\left(x;\theta \right)$ has mean zero and Fisher information as variance. This helps to establish the connection of Fisher information to the limiting distribution of maximum likelihood estimators, its additivity with respect to i.i.d. sample observations, and its role in the lower bounds of risk (information inequalities).

Wasserstein information [1] can be defined for the scalar parameter through the c.d.f. $F\left(x;\theta \right)=\int_{-\infty }^{x}p\left(x;\theta \right)d\theta$ as

$$W\left(\theta \right)=\int_{X}{\left(\frac{d}{d\theta }F\left(x;\theta \right)/p\left(x;\theta \right)\right)}^{2}\lambda \left(dx\right),$$

which does not require differentiablity of the density function p(x;θ). That opens new possibilities for the construction of an objective prior in the case of non-differentiable densities, see [2].

However, we are interested in even less regular situations (including uniform densities with support depending on parameter) for which neither Fisher information I(θ) nor Wasserstein information W(θ) can be helpful, while Hellinger information J(θ) may function as their substitute.

### 2.2. Information Inequalities

We define the quadratic Bayes risk for an estimator $\theta^{*}=\theta^{*}\left(X^{\left(n\right)}\right)$ constructed by an independent identically distributed sample $X^{\left(n\right)}=\left(X_{1},\ldots ,X_{n}\right)$ of size *n* from the model considered above with $p\left(x^{\left(n\right)};\theta \right)=\frac{dP_{\theta }^{\left(n\right)}}{d\lambda^{n}}$ and prior π(θ) as

$$R\left(\theta^{*}\right)=\int_{X^{n}}\int_{\Theta }{\left(\theta^{*}\left(x^{\left(n\right)}\right)-\theta \right)}^{2}p\left(x^{\left(n\right)};\theta \right)\pi \left(\theta \right)\lambda^{n}\left(dx\right)d\theta .$$

Let us consider an integral version of the classical Cramér–Frechet–Rao inequality, which under certain regularity conditions leads to the following asymptotic lower bound for the Bayes risk in terms of Fisher information:

(4) $$\underset{\theta^{*}}{\inf}R\left(\theta^{*}\right)\geq n^{-1}\int_{\Theta }I^{-1}\left(\theta \right)\pi \left(\theta \right)d\theta +O\left(n^{-2}\right).$$

This lower bound, which can be proven to be tight, was first obtained by [10], also in [11,12] under slightly different regularity assumptions. This bound can be extended to the nonregular case, when Fisher information may not exist. One of these extensions is Hellinger information inequality, providing an asymptotic lower bound

(5) $$\underset{\theta^{*}}{\inf}R\left(\theta^{*}\right)\geq n^{-2/\alpha }C\left(\alpha \right)\int_{\Theta }J^{-1}\left(\theta \right)\pi \left(\theta \right)d\theta +O\left(n^{-2/\alpha }\right)$$

obtained in [6] under the assumptions of Hellinger information J(θ) being strictly positive, almost surely continuous, bounded on any compact subset of Θ, and satisfying condition $\int_{\Theta }J^{-1}\left(\theta \right)\pi \left(\theta \right)d\theta <\infty$, where Θ is an open subset of real numbers and the constant C(α) is related to technical details of the proof and is not necessarily tight.

The key identity establishing the role of J(θ) in the case of i.i.d. samples $x^{\left(n\right)}=\left(x_{1},\ldots ,x_{n}\right)$

(6) $$h_{n}^{2}\left(\theta_{1},\theta_{2}\right)=\int_{X}{\left(\sqrt{p(x^{\left(n\right)};\theta_{1})}-\sqrt{p(x^{\left(n\right)};\theta_{2})}\right)}^{2}\lambda^{n}\left(dx\right)=2\left[1-{\left(1-\frac{1}{2}h^{2}\left(\theta_{1},\theta_{2}\right)\right)}^{n}\right]$$

easily follows from the definition and independence of $\left\{X_{i}\right\}$. Similar to the additivity of Fisher information, it allows for a transition from a single observation to a sample.

### 2.3. Hellinger Priors

The three most popular ways to obtain a non-informative prior might be as follows:

- The Jeffreys rule $\pi \left(\theta \right)\propto \sqrt{I(\theta )}$ [13],
- Probability matching priors [14,15],
- Reference priors [16,17].

For many regular parameter families, probability matching and reference priors both satisfy the Jeffreys rule. However, it is not necessary in case of multi-parametric families and the loss of regularity. Let us focus on the nonregular case, when Fisher information may not be defined. Most comprehensive results on reference priors in the nonregular case were obtained in [18].

Define Hellinger prior for the parametric set as in [4,8]:

(7) $$\pi_{H}\left(\theta \right)\propto \sqrt{J(\theta )}.$$

Hellinger priors will often coincide with well-known priors obtained by the approaches described above. However, there are some distinctions. A special role might be played by Hellinger priors in nonregular cases. We provide two simple examples of densities with support depending on the parameter.

**Example** **1.** *Uniform $X∼\mathrm{Unif}\left(\theta^{-1},\theta \right),\theta \in \left(1,\infty \right)$.*

$$\alpha =1,\pi_{H}\left(\theta \right)\propto J\left(\theta \right)=\frac{\theta^{2}+1}{\theta (\theta^{2}-1)}.$$

*The same prior can be constructed as the probability matching prior g or the reference prior [18].*

**Example** **2.** *Uniform $X∼\mathrm{Unif}\left(\theta ,\theta^{2}\right),\theta \in \left(1,\infty \right)$.*

(8) $$\alpha =1,\pi_{H}\left(\theta \right)\propto J\left(\theta \right)=\frac{2\theta +1}{\theta (\theta -1)}.$$

This prior is different from the reference prior obtained in [18] by rather technical calculations, maximizing the Kullback–Leibler divergence from the prior to the posterior:

(9) $$\pi_{R1}\left(\theta \right)\propto \frac{2\theta -1}{\theta (\theta -1)}\exp\left\{\psi \left(\frac{2\theta }{2\theta -1}\right)\right\},$$

where $\psi \left(z\right)=\frac{d}{dz}\log\Gamma \left(z\right),z>0$ is the polygamma function order 1. Tri Minh Le [19] used a similar approach, maximizing Hellinger distance between the prior and the posterior, and obtained

(10) $$\pi_{R2}\left(\theta \right)\propto \frac{{\left(2\theta +1\right)}^{2}{\left(2\theta -1\right)}^{5}}{\theta^{3}\left(\theta -1\right){\left[20\sqrt{\pi }{\left(2\theta -1\right)}^{2}-2{\left(2\theta +1\right)}^{2}\right]}^{2}}.$$

**Figure 1:**
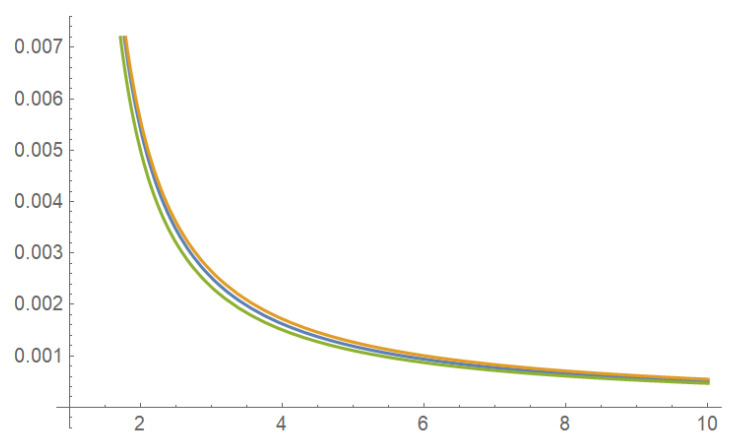
Three priors for Example 2: $\pi_{H}\left(\theta \right),\pi_{R1}\left(\theta \right),\pi_{R2}\left(\theta \right)$.

All three priors in (8), (9), and (10) have distinct functional forms. However, they are very close after appropriate re-normalization on the entire domain, which can be demonstrated graphically and numerically. See Figure 1 and the following comment.

For instance, the ratio $\pi_{H}\left(\theta \right)/\pi_{R2}\left(\theta \right)$ monotonically increases for θ from 1 to *∞* so that $0.996<\pi_{H}\left(\theta \right)/\pi_{R2}\left(\theta \right)<1.083.$

### 2.4. Optimal Design

A polynomial model of the experimental design may be presented as in [20],

$$y_{i}=\sum_{k=0}^{q}\theta_{k}x_{i}^{k}+\epsilon_{i},i=1,\ldots ,n,$$

where $x_{i}$ are scalars, θ is the unknown vector parameter of interest, and errors $\epsilon_{i}$ are non-negative i.i.d variables with density $p_{0}\left(y;\alpha \right)∼\alpha c\left(\alpha \right)y^{\alpha -1}$ (e.g., Weibull or Gamma). The space of balanced designs is defined as

$$\Xi =\{\xi =\left(w_{i},x_{i}\right),i=1,\ldots ,n:\sum_{i=1}^{n}w_{i}x_{i}=0,x_{i}\in \left[-A,A\right]\},$$

and there exist several definitions of optimal design. Lin et al. [7] suggest using criterion

$$\xi_{opt}=\arg\max_{\xi }J_{\xi }\left(\theta \right),J_{\xi }\left(\theta \right)=\underset{u}{\inf}J_{\xi }\left(\theta ,u\right)$$

where $J_{\xi }\left(\theta ,u\right)$ is Hellinger information in the direction $u\in R^{q+1}$, defined as

(11) $$\lim_{\epsilon \to 0}\frac{h(\theta ,\theta +\epsilon u)}{{\left|\epsilon \right|}^{\alpha }}=J_{\xi }\left(\theta ,u\right),$$

which is similar to the definition given in Section 1, but notice the difference with (2) in the treatment of powers: $\epsilon^{\alpha }$ versus $\epsilon^{2}$ in the denominator. Notice also that index of regularity α is assumed to be the same for all components of the vector parameter.

## 3. Hellinger Information Matrix for Vector Parameter

In this section, we concentrate on the multivariate parameter case allowing for different degrees of regularity for different components of the vector parameter. We define the Hellinger information matrix, determine our understanding of matrix information inequalities, formulate main results establishing lower bounds for the Bayes risk in terms of Hellinger information matrix, and provide examples of Hellinger priors illustrating the conditions of Theorems 1 and 2.

Proofs of the main results use the approach developed in [5]; Example 3 was previously mentioned in [8].

### 3.1. Definitions

Extending definitions of Section 1 to the vector case $\Theta \subset R^{m},m=1,2,\ldots$, we first introduce, as in [6], the *Hellinger distance matrix H* with elements

(12) $$H_{ij}\left(\theta ,U\right)=\int_{X}\left(\sqrt{p(x;\theta )}-\sqrt{p(x;\theta +u_{i})}\right)\left(\sqrt{p(x;\theta )}-\sqrt{p(x;\theta +u_{j})}\right)\lambda \left(dx\right)$$

where increments $u_{i}$ are columns of an m×m matrix *U*. Define also vectors $\alpha ={\left(\alpha_{1},\ldots ,\alpha_{m}\right)}^{\prime }$ (**index of regularity** with components $0<\alpha_{i}\leq 2$ ) and $\delta ={\left(\delta_{1},\ldots ,\delta_{m}\right)}^{\prime }$ with components $\delta_{i}=\epsilon^{2/\alpha_{i}}$, Δ=Diag(δ) such that for all i=1,…,m there exist finite non-degenerate limits

$$0<\lim_{\epsilon \to 0}{\left|\epsilon \right|}^{-2}H_{ii}^{2/\alpha_{i}}\left(\theta ,\Delta \right)<\infty$$

Then, the **Hellinger information matrix** will be defined by its components

(13) $$J_{ij}\left(\theta \right)=\lim_{\epsilon \to 0}{\left|\epsilon \right|}^{-2}H_{ij}^{1/\alpha_{i}+1/\alpha_{j}}\left(\theta ,\Delta \right).$$

Notice that components of the vector index of regularity α can be different, and therefore, components of the vector of increments δ can have different orders of magnitude with respect to ϵ. As a result, while the elements of matrix H(θ,Δ) may expose different local behavior depending on the components of α, the elements of matrix J(θ) are all finite.

### 3.2. Information Inequalities

Define the matrix of Bayes risk for an i.i.d. sample $x^{\left(n\right)}=\left(x_{1},\ldots ,x_{n}\right)$ with $p\left(x^{\left(n\right)};\theta \right)=\frac{dP_{\theta }^{\left(n\right)}}{d\lambda^{n}}$ as

(14) $$R\left(\theta^{*}\right)=\int_{X^{\left(n\right)}}\int_{\Theta }\left(\theta^{*}\left(x^{\left(n\right)}\right)-\theta \right){\left(\theta^{*}\left(x^{\left(n\right)}\right)-\theta \right)}^{\prime }p\left(x^{\left(n\right)};\theta \right)\pi \left(\theta \right)\lambda^{n}\left(dx\right)d\theta$$

and matrix ordering A(n)⪰B(n) as asymptotic positive semi-definite property

(15) $$\underset{|v|=1}{\inf}\sum_{i=1}^{m}\sum_{j=1}^{m}{\left(A\left(n\right)-B\left(n\right)\right)}_{ij}v_{i}v_{j}\geq -\delta_{n},\lim_{n\to \infty }\delta_{n}||A\left(n\right)-{B\left(n\right)||}^{-1}=0,\delta_{n}>0.$$

Let also

$$E\left(g(X^{\left(n\right)},\theta )\right)=\int_{X^{\left(n\right)}}\int_{\Theta }g\left(x^{\left(n\right)},\theta \right)p\left(x^{\left(n\right)};\theta \right)\pi \left(\theta \right)\lambda^{n}\left(dx\right)d\theta$$

denote the expectation over $\left(X^{\left(n\right)},\Theta \right)$. The following results formulate conditions under which the lower bounds for risk (14) in the sense of (15) are obtained in terms of Hellinger information and the index of regularity.

### 3.3. Main Results

**Theorem** **1.** *Let $\alpha ={\left(2,\ldots ,2,\alpha_{m}\right)}^{\prime }$, $\alpha_{m}\in \left[1,2\right)$,*

$$J_{ij}\left(\theta \right)=I_{ij}\left(\theta \right),i,j=1,\ldots ,m-1;J_{im}=0,J_{mm}\neq 0.$$

$$\left(\begin{array}{cccc} & & & 0\\ & I(\theta ) & & \ldots \\ & & & 0\\ 0 & \ldots & 0 & J_{mm}\left(\theta \right) \end{array}\right)$$

*Then, if $EI^{-1}\left(\theta \right)<\infty$, $EJ_{mm}^{-1}\left(\theta \right)<\infty$, it is true that*

1.
J(θ)>0

2.
$\inf_{\theta^{*}}R\left(\theta^{*}\right)⪰C_{1}\left(\alpha_{m}\right)Diag\left(n^{-2/\alpha_{m}}\right)EJ^{-1}\left(\theta \right)$

**Theorem** **2.** *Let $\alpha_{1}=\cdots =\alpha_{m}=1$,*

$$J_{ij}\left(\theta \right)=0,i\neq j;J_{ii}\neq 0.$$

$$\left(\begin{array}{cccc} J_{11}\left(\theta \right) & 0 & \ldots & 0\\ 0 & J_{22}\left(\theta \right) & 0 & \ldots \\ \ldots & 0 & \ldots & 0\\ 0 & \ldots & 0 & J_{mm}\left(\theta \right) \end{array}\right)$$

*Then, if $EJ_{ii}^{-1}\left(\theta \right)<\infty$, it is true that*

1.
J(θ)>0

2.
$\inf_{\theta^{*}}R\left(\theta^{*}\right)⪰C_{2}n^{-2}EJ^{-1}\left(\theta \right)$

Proofs of Theorems 1 and 2 are technically similar to the proof of the main results of [5], although the definition of Hellinger information was not explicitly provided in that paper.

### 3.4. Hellinger Priors

If J>0, as in the conditions of Theorems 1 and 2, the vector Hellinger prior can be defined as

$$\pi_{H}\left(\theta \right)\propto \sqrt{\det J(\theta )}.$$

In the case of all components $\alpha_{i}\equiv 2$, Hellinger information reduces to the Fisher information matrix, and our approach leads to the Jeffreys prior [12].

**Example** **3.** *Truncated Weibull distribution (see Theorem 1):*

$$p\left(x;\beta ,\phi ,\tau \right)=\beta \phi^{\beta }x^{\beta -1}\exp\left\{-\phi^{\beta }\left(x^{\beta }-\tau^{\beta }\right)\right\},x\in \left[\tau ,\infty \right)$$

**Figure 2:**
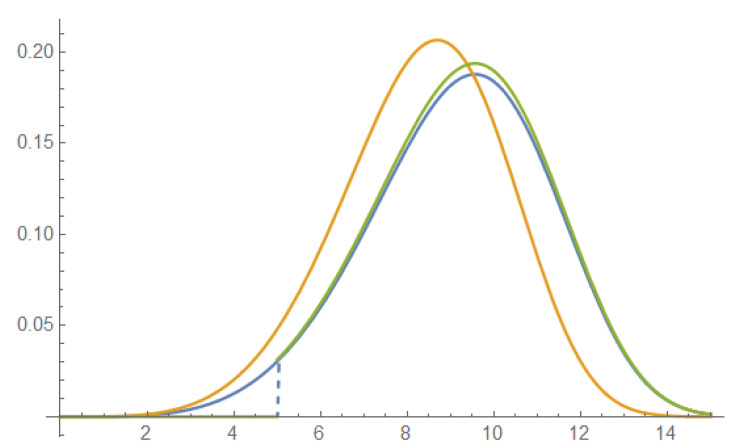
Truncated Weibull p(x; 5, 0.1, 0); p(x; 5, 0.11, 0); p(x; 5, 0.1, 5).

*with two parameters of interest: regular pseudo-scale ϕ>0 and nonregular threshold τ>0. Assume β>2 fixed. See Figure 2.*

Using notation θ=(ϕ,τ), obtain $\alpha_{1}=2,\alpha_{2}=1,\delta_{1}=\epsilon ,\delta_{2}=\epsilon^{2}$ and

$$H\left(\theta ,\Delta \right)=\left(\begin{array}{cc} \delta_{1}\phi^{-2} & o(\delta_{1}\delta_{2})\\ o(\delta_{1}\delta_{2}) & \delta_{2}\phi^{2\beta }\tau^{2\beta -2} \end{array}\right).$$

After limit transition in δ,

$$J\left(\theta \right)=\left(\begin{array}{cc} \phi^{-2} & 0\\ 0 & \phi^{2\beta }\tau^{2\beta -2} \end{array}\right).$$

Therefore, $\pi_{H}\left(\theta \right)\propto \phi^{\beta -1}\tau^{\beta -1}$, which is also the reference prior for the vector parameter [21].

**Example** **4.** *Circular beta distribution on a disc (see Theorem 1):*

$$p\left(r,w;\beta_{1},\beta_{2},\rho \right)=\frac{r}{\pi \rho^{2}}{\left(\frac{w}{2\pi }\right)}^{\beta_{1}-1}{\left(1-\frac{w}{2\pi }\right)}^{\beta_{1}-1}$$

**Figure 3:**
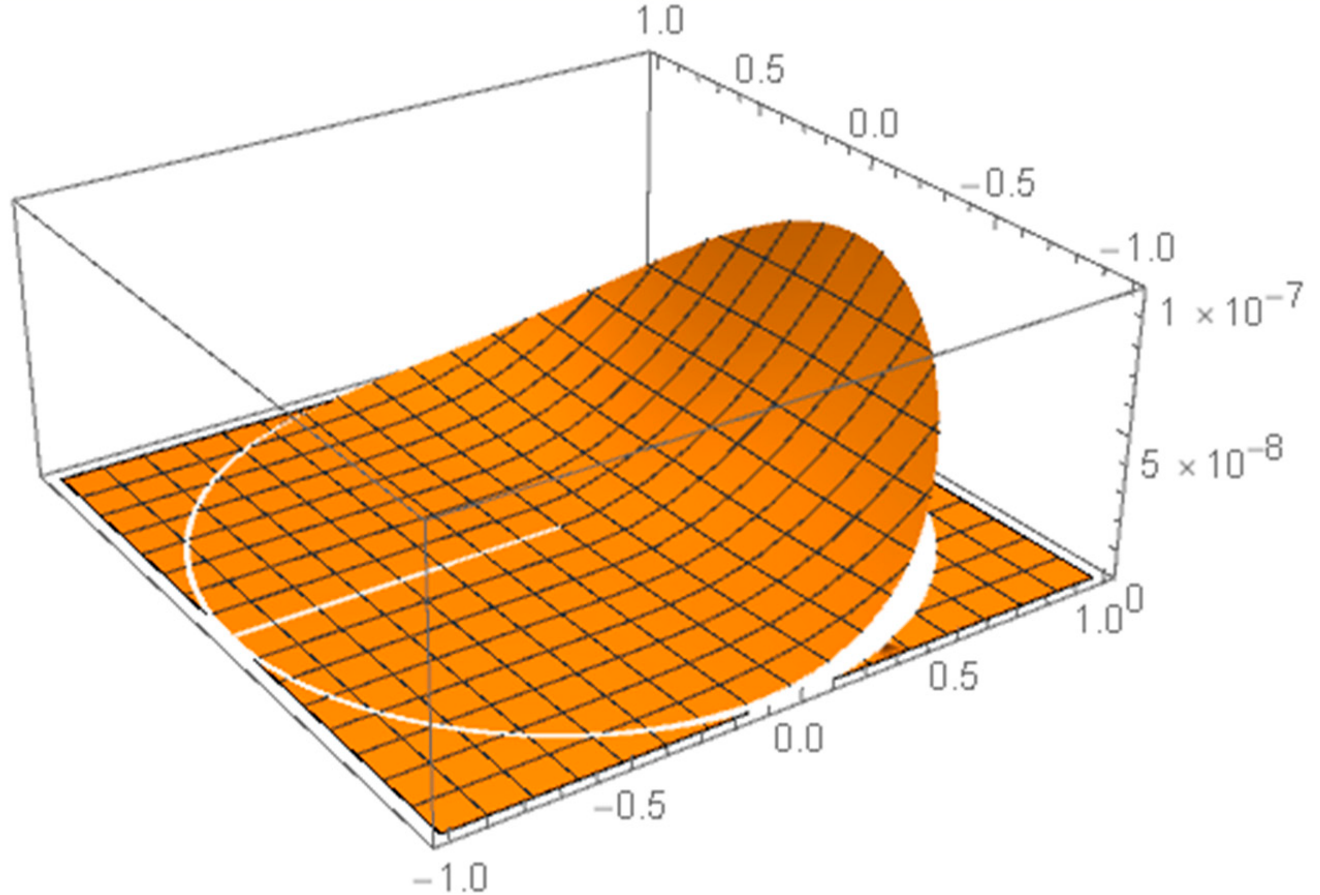
Truncated beta on a disc.

*with three parameters of interest: regular $\beta_{1},\beta_{2}>1$ and nonregular radius ρ>0. See Figure 3.*

$$J\left(\theta \right)=\left(\begin{array}{ccc} \psi \left(\beta_{1}\right)-\psi \left(\beta_{1}+\beta_{2}\right) & -\psi (\beta_{1}+\beta_{2}) & 0\\ -\psi (\beta_{1}+\beta_{2}) & \psi \left(\beta_{1}\right)-\psi \left(\beta_{1}+\beta_{2}\right) & 0\\ 0 & 0 & 4\rho^{-2} \end{array}\right),$$

where ψ(z) is the polygamma function of order 1.

Therefore, $\pi_{H}\left(\theta \right)\propto \rho^{-1}\sqrt{\psi \left(\beta_{1}\right)\psi \left(\beta_{2}\right)-\psi \left(\beta_{1}+\beta_{2}\right)\left[\psi \left(\beta_{1}\right)+\psi \left(\beta_{2}\right)\right]}$

**Example** **5.** *Uniform on a rectangle (see Theorem 2): $X∼\mathrm{Unif}\left(\theta ,\theta^{2}\right),\theta \in \left(1,\infty \right)$ with two parameters of interest: regular pseudo-scale ϕ>0 and nonregular threshold* τ>0.

*Using notation $\theta =(\theta_{1},\theta_{2})$, obtain $\alpha_{1}=\alpha_{2}=1,\delta =\delta_{1}=\delta_{2}=\epsilon^{2}$ and*

$$H\left(\theta ,\Delta \right)=\left(\begin{array}{cc} \delta \theta_{1}^{-2} & \delta^{2}{\left(\theta_{1}\theta_{2}\right)}^{-1}\\ \delta^{2}{\left(\theta_{1}\theta_{2}\right)}^{-1} & \delta \theta_{2}^{-2} \end{array}\right).$$

*After the limit transition,*

$$J\left(\theta \right)=\left(\begin{array}{cc} \theta_{1}^{-2} & 0\\ 0 & \theta_{2}^{-2} \end{array}\right).$$

*Therefore, $\pi_{H}\left(\theta \right)\propto \theta_{1}^{-1}\theta_{2}^{-1}$, also the reference prior.*

**Example** **6.** *Uniform with two moving boundaries $X∼\mathrm{Unif}\left(\theta_{1},\theta_{1}+\theta_{2}\right),\theta_{2}\in \left(0,\infty \right)$ (neither Theorem 1 nor Theorem 2 applies): using notation $\theta =(\theta_{1},\theta_{2})$, obtain $\alpha_{1}=\alpha_{2}=1,\delta =\delta_{1}=\delta_{2}=\epsilon^{2}$ and*

$$H\left(\theta ,\Delta \right)=\left(\begin{array}{cc} 2\delta \theta_{2}^{-2} & \delta \theta_{2}^{-2}\\ \delta \theta_{2}^{-2} & \delta \theta_{2}^{-2} \end{array}\right).$$

*After limit transition,*

$$J\left(\theta \right)=\left(\begin{array}{cc} 2\theta_{2}^{-2} & \theta_{2}^{-2}\\ \theta_{2}^{-2} & \theta_{2}^{-2} \end{array}\right).$$

*Therefore, $\pi_{H}\left(\theta \right)\propto \theta_{2}^{-2}$, also the reference prior.*

## 4. Discussion

A Hellinger information matrix can be defined in a reasonable way to serve as a substitute for the Fisher information matrix in multivariate nonregular cases. It can be used as a technically simple tool for the elicitation of non-informative priors.

Properties of the Hellinger distance (symmetry, etc.) grant certain advantages vs analogous constructions based on Kullback–Leibler divergence.

Some interesting nonregularities are not covered by the conditions of Theorems 1 and 2 (see Example 6). More general results related to a positive definite property of J(θ) would be interesting.

It is tempting to obtain Hellinger priors as the solution of a particular optimization problem (similar to the reference priors).

## Data Availability

No new data were created or analyzed in this study. Data sharing is not applicable to this article.
